# Mechanism of Huaiqihuang (HQH) against cyclophosphamide (CYP)-induced hippocampal neurotoxicity based on network pharmacology, molecular docking and experimental verification

**DOI:** 10.3389/fcell.2025.1629110

**Published:** 2025-09-03

**Authors:** Yueming Zhang, Fengwei Huang, Jinghui Zhai, Jingmeng Sun, Boyu Li, Sixi Zhang

**Affiliations:** ^1^ Department of Clinical Pharmacy, The First Hospital of Jilin University, Changchun, China; ^2^ School of Pharmacy, Jilin University, Changchun, China; ^3^ Second Clinical Medical College, Harbin Medical University, Harbin, China

**Keywords:** Huaiqihuang, cyclophosphamide, neurotoxicity, network pharmacology, molecular docking

## Abstract

**Background:**

Cyclophosphamide (CYP) is widely used for the treatment of cancer and autoimmune diseases. However, neurotoxicity accompanied with application of CYP seriously affects the final clinical outcome. Huaiqihuang (HQH) is a Chinese herbal complex with immunomodulatory effect and widely used for treating various diseases. The present research was conducted to evaluate the protective effect of HQH against CYP-induced neurotoxicity and to elucidate the underlying mechanisms.

**Methods:**

Sprague–Dawley rats were randomly divided into four groups (10 per group): the CYP-only group (single dose of 200 mg/kg), low- and high-dose HQH + CYP groups (pretreatment with 3 or 6 g/kg HQH for 5 days), and control (saline) group. Histopathological analysis and behavioral tests was used to evaluate the therapeutic effects of HQH on CYP-induced neurotoxicity. Network pharmacology, molecular docking, and Western blot were employed to assess the anti-neurotoxicity mechanisms.

**Results:**

Both doses of HQH restored histopathological aberrations, oxidative stress and inflammation caused by CYP in rats. Behavioral tests showed that HQH pretreatment improved motor coordination and balance in CYP-treated rats. Network pharmacology identified core targets including HSP90AA1, TP53, MAPK1, AKT1, RELA, TNF. Molecular docking revealed that TNF, HSP90AA1, TP53, and MAPK1 had strong binding affinities with CYP. Experimental validation using Western blot confirmed that HQH significantly decreased the protein expression of TNF, HSP90AA1, TP53, and MAPK1 in hippocampal tissues.

**Conclusion:**

HQH mitigates CYP-induced hippocampal neurotoxicity by decreasing oxidative stress, and inflammation, with HSP90AA1 being a key target, providing a novel therapeutic strategy for chemotherapy-associated cognitive impairment.

## 1 Introduction

Cyclophosphamide (CYP), a widely used alkylating agent since the 1950s, remains indispensable in the treatment of malignancies and autoimmune disorders. However, its clinical utility is limited by dose-limiting neurotoxicity, which manifests as hippocampal neurodegeneration, cognitive decline, and memory deficits—a condition collectively referred to as “chemobrain” (Hussein et al., 2024; H M Ibrahim and Elnaggar, 2020; S and Kumar, 2019).

The hippocampus, a critical brain region for learning and memory, is especially vulnerable to CYP-induced damage due to its high sensitivity to oxidative stress, inflammation, and apoptosis (H M Ibrahim and Elnaggar, 2020; Zhaoping Ou et al., 2021). Mechanistically, CYP metabolism generates acrolein, a neurotoxic metabolite that disrupts redox homeostasis through ROS overproduction, leading to mitochondrial dysfunction (evidenced by cristaeolysis and ER dilation) (H M Ibrahim and Elnaggar, 2020), and suppression of Nrf2-mediated antioxidant defenses coupled with NF-κB-driven neuroinflammation (Zhaoping Ou et al., 2021). These pathological processes lead to a 40%–60% reduction in hippocampal CA1/CA3 neuronal viability, accompanied by astrogliosis, and caspase-3-dependent apoptosis (H M Ibrahim and Elnaggar, 2020; S and Kumar, 2019), ultimately impairing therapeutic outcomes (Hussein et al., 2024). These findings underscore the urgent need for novel neuroprotective agents that can target oxidative stress, apoptosis, and inflammation to safeguard neural integrity and improve therapeutic outcomes in chemotherapy patients.

Traditional Chinese medicine (TCM) has shown potential efficacy in treating CYP-induced neurotoxicity (Chen et al., 2019). TCM has a long-standing history of addressing various neurological disorders, owing to its multi-faceted therapeutic properties. Huaiqihuang (HQH), a proprietary Chinese medicine composed of *(Trametes, Fructus Lycii, and Polygonatum)*, has demonstrated several beneficial effects, including antioxidant, anti-apoptotic, anti-inflammatory, and immunoregulatory activities (Li et al., 2025; Tsunenaga et al., 2022; Li et al., 2025; Wang et al., 2020). These properties have shown therapeutic efficacy in conditions such as asthma, arthritis, and nephrotic syndrome (Liang et al., 2017; T et al., 2019; Lin et al., 2021). Our previous study has indicated that HQH could alleviate CYP-induced nephrotoxicity (Zhang et al., 2021); however, its role in CYP-induced hippocampal neurotoxicity remained unknown. Given the extensive pharmacological activities of HQH and the pathophysiology of CYP-induced neurotoxicity, we hypothesize that HQH may provide neuroprotective effects against CYP-induced hippocampal damage.

Despite its long history of clinical use, the active components, target genes, and mechanisms underlying HQH’s protective effects against CYP-induced neurotoxicity remain poorly understood. Due to the complex composition of TCM, traditional pharmacological approaches struggle to fully elucidate its mechanisms. However, with the advent of network pharmacology, a novel approach has emerged to systematically explore the interactions between TCM, compounds, targets, signaling pathways, and diseases. Network pharmacology offers a powerful method for predicting and analyzing the multi-target and multi-pathway mechanisms of TCM (Deng et al., 2024). Molecular docking, a key tool in virtual screening and drug discovery, also plays an important role in identifying protein-ligand interactions, aiding in drug design (Tingting Chen et al., 2022).

**FIGURE 1:**
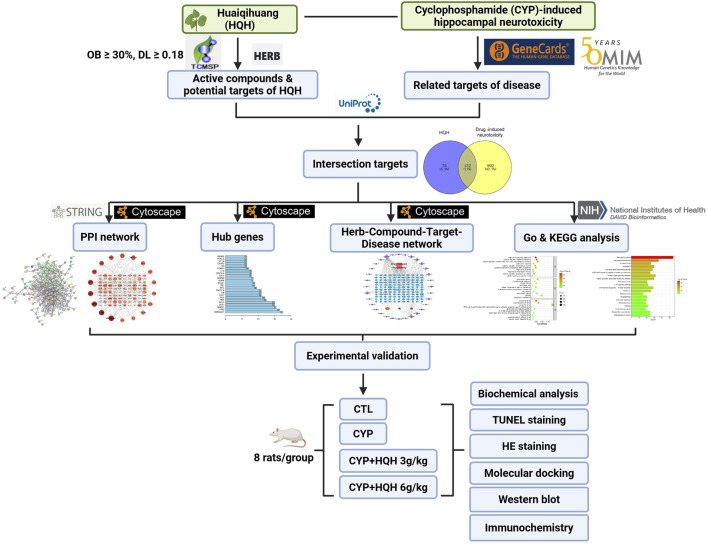
The workflow of the study.

In this study, we aim to investigate HQH’s mechanism of action against CYP-induced neurotoxicity using network pharmacology and molecular docking. First, we will predict potential active ingredients, target genes, and signaling pathways of HQH. Second, the most reliable candidate components, target genes, and pathways will be experimentally validated *in vitro* and *in vivo*. The overall study design is outlined in Figure 1.

## 2 Materials and methods

### 2.1 Animals and drugs

Fifty male Sprague–Dawley rats (200 ± 20 g; 6 weeks old) obtained from the Experimental Animal Center of Jilin University (Jilin, China) were housed in plastic cages at temperature (22 °C ± 3 °C) and humidity (50% ± 10%) with a 12-h/h light/dark circle. All animal handling procedures deferred to the National Institutes of Health Guide (NIH Publication no. 85-23, revised 2011). The experimental scheme achieved support from the Animal Ethics Committee of Changchun University of Chinese Medicine. Food and drink were provided *ad libitum*. After acclimatized for 7 days, rats were randomly divided into the following six groups (n = 10/group), CTL group, CYP (200 mg/kg, i. p.)-only group, low-dose HQH (3 g/kg HQH, i. g.)+CYP group, high-dose HQH (6 g/kg, i. g.) (Zhang et al., 2021) +CYP, HQH (6 g/kg, i. p.)-only group, 17-DMAG (HSP90 inhibitor, 5 mg kg^-1^, i. p.) (Hu et al., 2023)+CYP group, Pifithrin-α (TP53 inhibitor, 10 mg/kg, i. p.) (Xu et al., 2025)+CYP group. HQH, 17- DMAG and Pifithrin-α were administered once daily for 5 consecutive days. On the 5^th^ day, CYP was administered 30 min after HQH treatment. 1 d after the last dose of CYP, the animals were carefully anesthetized and sacrificed. Prior to anesthesia, animals were fasted for 12 h to minimize the risk of aspiration. Anesthesia was maintained using isoflurane (1.5%–2% in oxygen) delivered via a precision vaporizer. Blood samples were collected from abdominal aorta to separate serum for the assessment of malondialdehyde (MDA), superoxide dismutase (SOD), and catalase (CAT) activity. After the blood is taken, brain tissues were mildly removed for further biochemical analysis. To ensure the humane treatment of animals during experiments, a humane endpoint protocol was established, which included the administration of 5% isoflurane via inhalation for a minimum of 5 min, followed by cervical dislocation.

### 2.2 Histological examination

The brain tissue was washed by saline and fixed in 4% formalin for 48 h, then sliced coronally to get hippocampal plane. After embedded in paraffin wax, the hippocampal plane was cut into 5-mm sections and stained with hematoxylin and eosin (H&E). The pathologist who was blind to the treatment groups was employed to assess the slice. The sections were observed at ×100 and ×400 magnification with a light microscope (Nikon Eclipse TE2000-U, Nikon, Japan) to estimate the changes of NLRP3.

### 2.3 Biochemistry assays

The activities of catalase (CAT), glutathione (GSH), superoxide dismutase (SOD), and malondialdehyde (MDA) in serum were determined using diagnostic kits (Nanjing Jiancheng Bioengineering Institute, Nanjing, China).

### 2.4 Screening of active compounds and target prediction of HQH

The active compounds of HQH were retrieved from the TCMSP database (https://www.tcmsp-e.com/tcmsp.php) using oral bioavailability (OB) ≥ 30% and drug-likeness (DL) ≥ 0.18 as selection criteria. For components not included in TCMSP, the HERB database (http://herb.ac.cn/) was used for further identification (Ru et al., 2014). The potential targets associated with the identified compounds were extracted from TCMSP, and further standardized using the UniProt database (https://www.uniprot.org/), ensuring consistency in gene and protein names. Duplicates were removed to obtain a comprehensive list of candidate targets for HQH.

### 2.5 Identification of targets associated with CYP-induced hippocampal neurotoxicity

Potential disease-associated targets were retrieved by searching for “drug-induced neurotoxicity” in the OMIM (https://omim.org/) and Genecards (https://www.genecards.org/) databases. Results from both databases were combined, and duplicate entries were removed to generate a refined set of targets associated with CYP-induced hippocampal neurotoxicity.

### 2.6 Identification of intersectional targets and construction of the PPI network

The intersection of HQH-related targets and disease-associated targets was determined using the Venny 2.1.0 tool ((https://bioinfogp.cnb.csic.es/tools/venny/), and a Venn diagram was generated to illustrate the overlap. To further understand the protein-protein interactions (PPIs) among these targets, the STRING database (version 11.5) (https://string-db.org/) was used, setting the species to *Homo sapiens* and an interaction confidence threshold of 0.9 (highest reliability) (Szklarczyk et al., 2021). Isolated nodes were systematically excluded to enhance network biological relevance. The resulting PPI network was visualized and analyzed using Cytoscape (version 3.9.1). The degree centrality of each node was calculated to determine the key targets. Core targets were identified based on their degree values, and the top 20 hub genes were selected and visualized using R (version 4.2.1). Additionally, MCODE clustering analysis in Cytoscape was performed to identify significant gene clusters, highlighting core genes based on maximal cluster centrality scores.

### 2.7 Construction and analysis of the Herb-Compound-Target-Disease network

A multi-layered interaction network integrating HQH active ingredients, intersection targets, and disease-associated biological components was constructed using Cytoscape 3.9.1. Isolated nodes lacking direct interactions were filtered out to enhance biological relevance. Network topology was analyzed using the Network Analyzer tool, and node importance was ranked based on the degree metric.

### 2.8 GO analysis and KEGG signaling pathway

The intersection targets were uploaded to the DAVID database (https://david.ncifcrf.gov/) for GO biological process and KEGG pathway enrichment analysis (p < 0.05). GO functional analysis was categorized into Biological Process (BP), Molecular Function (MF), and Cellular Component (CC), while KEGG pathway analysis was performed to identify significantly enriched signaling pathways related to HQH’s mechanism of action. The results of GO and KEGG enrichment analyses were visualized as bar plots and bubble charts using R (version 4.2.1).

### 2.9 Molecular docking

To evaluate potential interactions between HQH compounds and core targets, molecular docking was conducted using AutoDock Vina (version 1.1.2). The 3D structures of key active compounds were obtained from the PubChem database (https://pubchem.ncbi.nlm.nih.gov/) in SDF format and processed using Chem3D for energy minimization via the MM2 force field module. The optimized compounds were saved in MOL2 format. Using AutoDockTools (version 1.5.6), hydrogen atoms and Gasteiger charges were added, rotatable bonds were defined, and the ligands were converted to PDBQT format. The crystal structure of AKT1 (PDB ID: 2UVM), HSP90AA1 (PDB ID: 3O0I), MAPK1(PDB ID: 8AO6), RELA (PDB ID: 1NFI), TNF(PDB ID:5M2J), TP53(PDB ID:1C26) was obtained from the Protein Data Bank (http://www.rcsb.org) and processed in PyMOL to remove non-essential water molecules and heteroatoms. The AutoDockTools software was used to add polar hydrogens and Gasteiger charges, and the processed protein structures were saved in PDBQT format. Molecular docking was performed using AutoDock Vina, with binding conformations ranked based on binding affinity (kcal/mol). The top-ranked docking poses were visualized using PyMOL.

### 2.10 Western blotting analysis

Total protein was prepared as previously reported. Briefly, the tissues and cells were washed by PBS and then solubilized by radioimmunoprecipitation assay (RIPA) lysis buffer enhanced with 1% phenylmethanesulfonyl fluoride. Bicinchoninic acid (BCA) assay was used to assess the protein concentration. Western blotting was performed with 8% or 12% precast gels and then the separated protein was electrophoretically transferred to polyvinylidene fluoride membranes. The membranes were first blocked with 5% skim milk powder at room temperature for 1 h and then incubated at 4 °C overnight with primary antibody and β-actin. The membranes were then washed three times with TBST and incubated with secondary antibody (1:1,000) at room temperature for 1 h. After discarding the secondary antibody and washing three times with TBST, the target proteins were examined using an enhanced chemiluminescence reagent (Yeasen Biotech, Shanghai, China) and quantified using ImageJ software.

### 2.11 Statistical analysis

The data are expressed as mean ± standard deviation (SD). The differences between groups were assessed using Student’s t-test or one-way analysis of variance (ANOVA) followed by Dunnett’s test. *P* < 0.05 indicates statistical significance. The experiments were repeated at least three times.

## 3 Results

### 3.1 HQH reduces damage in CYP-treated rat hippocampus

**FIGURE 2:**
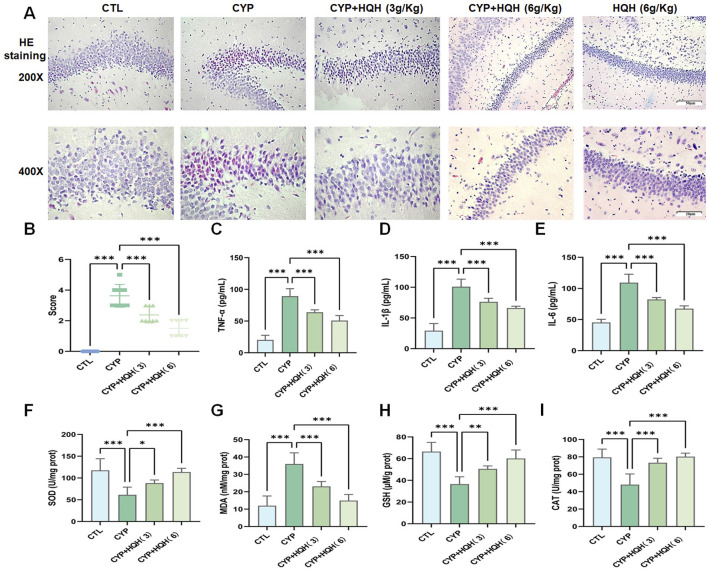
Huaiqihuang (HQH) Ameliorates Cyclophosphamide (CYP)-Induced Hippocampal Neurotoxicity. **(A)** Representative images of hippocampal sections stained with hematoxylin and eosin (H&E) at ×200 and ×400 magnification. **(B)** Beam Walking Test. **(C–E)** The levels of inflammatory cytokines (TNF-α, IL-1β, (IL-6) in hippocampal tissues. **(F–I)** The levels of antioxidant enzyme activity (SOD, GSH, CAT) and lipid peroxidation marker (MDA). Data are presented as mean ± SD, and statistical significance is indicated by asterisks: *p < 0.05, **p < 0.01, ***p < 0.001.

To explore the protective effects of HQH against CYP-induced neurotoxicity, 24 h after the final treatment, H&E staining was conducted to assess the histopathological changes in the hippocampus. The hippocampus architecture was normal in the control group, whereas the CYP administration resulted in obvious pathological alterations including disorganization, degeneration and necrosis of nerve cells, which were clearly rescued by HQH pretreatment. Notably, administration of HQH alone did not induce any changes in the hippocampus (Figure 2A). In addition, the beam-walking test, a behavioral assessment used to evaluate motor coordination and balance. The results indicate that the CYP group exhibited significantly impaired performance compared to the CTL group, as evidenced by higher beam walking scores. However, the CYP + HQH groups demonstrated significant improvement in performance. These findings suggest that HQH may have a beneficial role in mitigating the motor impairments associated with CYP treatment (Figure 2B). What’s more, the levels of tumor necrosis factor alpha (TNF-α), interleukin-1 beta (IL-1β), and interleukin-6 (IL-6) were measured as key indicators of inflammation. The CYP group exhibited significantly elevated levels of these pro-inflammatory cytokines compared to the CTL group, indicating a robust inflammatory response triggered by CYP treatment. In contrast, the CYP + HQH groups showed a dose-dependent decrease in the levels of TNF-α, IL-1β, and IL-6, suggesting that HQH possesses anti-inflammatory properties (Figures 2C–E). To further estimate the role of HQH in CYP-treated rats, the level of the endogenous lipid peroxidation marker MDA and the anti-oxidant activities of SOD, GSH and CAT in serum of CP-treated rats were detected. The MDA level was significantly higher and the SOD, GSH and CAT activities were significantly reduced in the CYP-only group compared to the control group. However, HQH pretreatment ameliorated these changes compared to the CYP-only group (Figures 2F–I). These findings imply that HQH may mitigate the hippocampus tissue damage induced by CYP, highlighting its potential as a therapeutic agent to counteract inflammation-related side effects of chemotherapy.

### 3.2 HQH reduces apoptosis in CYP-treated rat hippocampus

**FIGURE 3:**
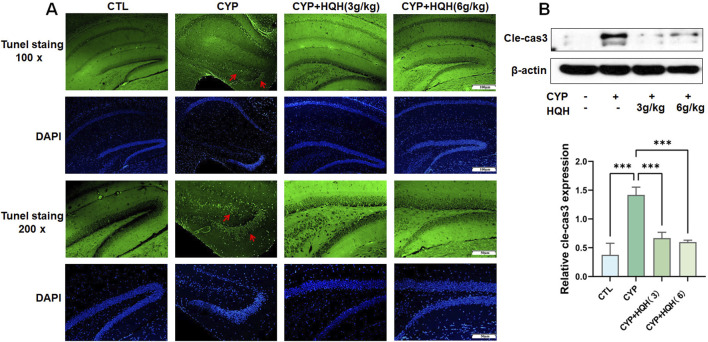
HQH Ameliorates CYP-induced Hippocampal apoptosis. **(A)** Representative images of hippocampal sections stained with TUNEL at ×100 and ×200 magnification. **(B)** The level of cleaved-caspase3 in hippocampal tissues detected by Western blot. Data are presented as mean ± SD, and statistical significance is indicated by asterisks: ***p < 0.001.

To explore whether the protective effect of HQH on CYP-treated rats was related to apoptosis, TUNEL staining and the apoptosis marker caspase-3 was determined. As expected, the control group (CTL) exhibited minimal apoptosis as indicated by the low number of TUNEL-positive cells. In contrast, the cyclophosphamide (CYP) treatment group displayed a significant increase in TUNEL-positive cells, highlighting the pro-apoptotic effect of CYP, the addition of HQH at both 3 g/kg and 6 g/kg doses led to a decrease in TUNEL-positive cells, suggesting that HQH can attenuate CYP-induced apoptosis (Figure 3A). Figure 3B illustrates the expression levels of cleaved-caspase 3 (Cle-cas3), a key executioner caspase in the apoptosis pathway, as determined by Western blot analysis. CYP significantly enhanced the expression of cleaved caspase-3 in hippocampus tissues, and these changes were significantly reversed by HQH. However, HQH pretreatment observably reversed these effects. The findings indicated that HQH inhibited CYP-induced apoptosis via regulating the expression of caspase-3.

### 3.3 Identification of active compounds and target networks in HQH for CYP-Induced neurotoxicity protection

**FIGURE 4:**
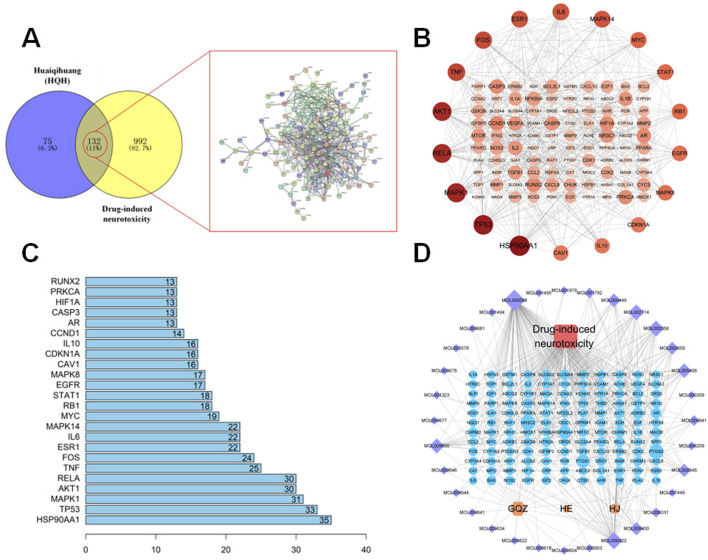
**(A)** Venn diagram illustrating overlapping targets (n = 132) between HQH-related targets (purple) and CYP-induced neurotoxicity-related targets (yellow). **A** corresponding PPI network is shown. **(B)** Protein-protein interaction (PPI) network of the 132 overlapping targets constructed via STRING and visualized using Cytoscape. **(C)** Bar chart representing the top 20 hub genes ranked by degree value from the PPI network. **(D)** Herb-Compound-Target-Disease Network. Yellow nodes represent HQH herbs (mainly includes GQZ: Gouqizi (*Lycium barbarum*); HE: Huaier (*Trametes*); HJ: Huangjing (*Polygonatum*)), purple nodes represent active compounds, blue nodes are target genes, and the red node indicates the disease. Node size varies based on degree value.

The TCMSP and HERB databases were used to screen the active compounds of HQH based on the pharmacokinetic parameters OB ≥ 30% and DL ≥ 0.18. After removing duplicates, 57 bioactive compounds were identified (Supplementary Table S1). The corresponding 207 target proteins were retrieved from the TCMSP database, and gene names were standardized using UniProt (UniProt Consortium, 2023). To identify potential targets associated with CYP-induced hippocampal neurotoxicity, we searched the GeneCards and OMIM databases using the keyword “drug-induced neurotoxicity.” After removing duplicates, a total of 1,124 disease-related targets were obtained (1,122 from GeneCards and 2 from OMIM). A total of 132 overlapping targets were identified between HQH-related targets (207) and disease-associated targets (1,124) using the Venny 2.1.0 tool. These targets represent potential mechanisms by which HQH may exert protective effects against drug-induced neurotoxicity. The STRING database was used to construct a PPI network based on these 132 intersection targets. The network consisted of 118 nodes and 490 edges, with connectivity analyzed using Cytoscape 3.9.1 (Figures 4A,B) (Shannon et al., 2003). Node size and color intensity represent degree values, with higher values indicating greater network connectivity. Notably, HSP90AA1, TP53, MAPK1, RELA, AKT1, and TNF emerged as central nodes within this network. These six targets exhibited high-degree values, signifying their substantial connections with other nodes and underscoring their potential significance in the pathophysiology of drug-induced neurotoxicity (Figure 4C). Subsequently, we used the Network Analyzer tool in the Cytoscape 3.9.1 to analyze the topology parameters of each target. The top 20 are selected as the core targets according to the ranking of Degree value, followed by HSP90AA1, TP53, MAPK1, AKT1, RELA, TNF, etc. To visualize the relationship between HQH components, target genes, and drug-induced neurotoxicity related pathways, a Herb-Compound-Target-Disease network was constructed in Cytoscape 3.9.1. The network originally contained 57 active compounds and 132 target genes, but 25 isolated compounds (lacking target interactions) were removed. The final network consisted of 32 effective HQH components (Supplementary Table S2, Supplementary Digital Content) interacting with 132 drug-induced neurotoxicity related targets (Figure 4D). Among these, cyanin, kaempferol, and baicalein emerged as key bioactive components with high-degree connectivity, suggesting their central roles in HQH’s therapeutic effects.

### 3.4 GO analysis and KEGG signaling pathway

**FIGURE 5:**
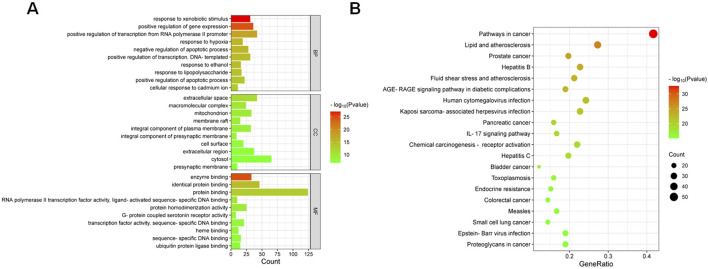
**(A)** GO enrichment analysis of the 132 overlapping targets. Top 10 terms from biological processes (BP), cellular components (CC), and molecular functions (MF) are shown. Red indicates higher significance (-log10 P-value). **(B)** KEGG pathway enrichment analysis of HQH’s core targets (top 20 pathways). Red indicates higher significance (-log10 P-value).

To explore the potential mechanisms, we imported 132 intersection targets into DAVID database for GO and KEGG pathway enrichment analysis (Sherman et al., 2022). A total of 1017 GO functional items were obtained, including 767 biological process (BP), which mainly involves response to xenobiotic stimulus, positive regulation of gene expression, positive regulation of transcription from RNA polymerase II promoter, and so on. There were 91 cellular component (CC), mainly involved extracellular space, macromolecular complex, mitochondrion, membrane raft, and so on. It also included 159 molecular function (MF), focusing on enzyme binding, identical protein binding, protein binding, RNA polymerase II transcription factor activity, etc. As shown in Supplementary Tables S3–S5, the first 10 items of enrichment results were visualized according to *p*-value (Figure 5A). We screened 170 major signaling pathways in KEGG pathway enrichment results. The most significant enriched 20 pathways in KEGG analysis were shown in Supplementary Table S6. The pathways with the highest enrichment levels included IL-17 signaling pathway, the core targets related to it included HSP90AA1, CXCL8, MMP1, MMP3, FOS, PTGS2, MAPK14, TNF, MMP9, RELA, NFKBIA, CXCL10, IL6, MAPK8, CASP8, IFNG, IL1B, CASP3, CCL2, and MAPK1. The top 20 potential signaling pathways were presented as bar chart (Figure 5B).

### 3.5 Molecular docking

**FIGURE 6:**
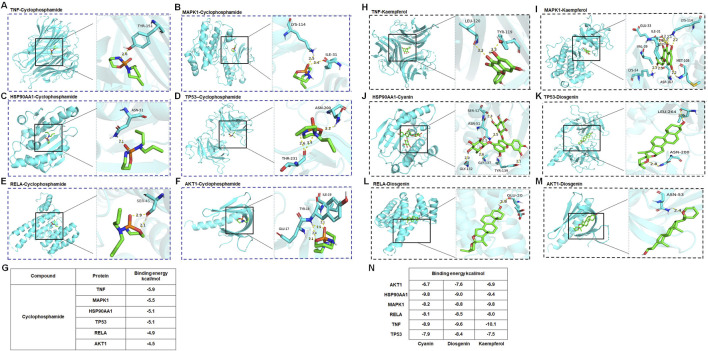
**(A–G)** Molecular docking of cyclophosphamide with key targets TNF, MAPK1, HSP90AA1, TP53, RELA, AKT1. **(H)** Molecular docking of kaempferol with TNF. **(I)** Molecular docking of kaempferol with MAPK1. **(J)** Molecular docking of cyanin with key HSP90AA1. **(K)** Molecular docking of diosgenin with TP53. **(L)** Molecular docking of diosgenin with RELA. **(M)** Molecular docking of diosgenin with AKT1. **(N)** The binding energies of compounds (Cyanin, Diogenin, Kaempferol) with six target proteins (AKT1, HSP90AA1, MAPK1, RELA, TNF, TP53).

In the process of molecular docking, the affinity between ligands and receptors is typically gauged by the binding energy using AutoDock Vina 1.1.2. The binding energy was calculated to evaluate the degree of complementarity between the component and the protein. A more negative binding energy value signifies a stronger binding interaction. In general, the binding energy lower than −5.00 kcal/mol indicated strong binding energy (Li et al., 2023). As depicted in Figures 6A–G, the binding energy of cyclophosphamide with six targets ranged from −5.95 to −4.5 kcal/mol, of which, four targets (TNF, HSP90AA1, TP53, MAPK1) with binding energy below −5 kcal/mol were identified as having high affinity and were thus considered key targets. These interactions may be crucial for its therapeutic effects. Additionally, Figures 6H–N shows high binding interactions between the active components (cyanin, kaempferol, diosgenin) of HQH granules and these six proteins, providing further insight into their potential as therapeutic agents. The comprehensive analysis of both the granules’ components and cyclophosphamide with the proteins has led to the selection of four targets as the most promising key targets for further investigation and potential therapeutic application.

### 3.6 Experimental verification

**FIGURE 7:**
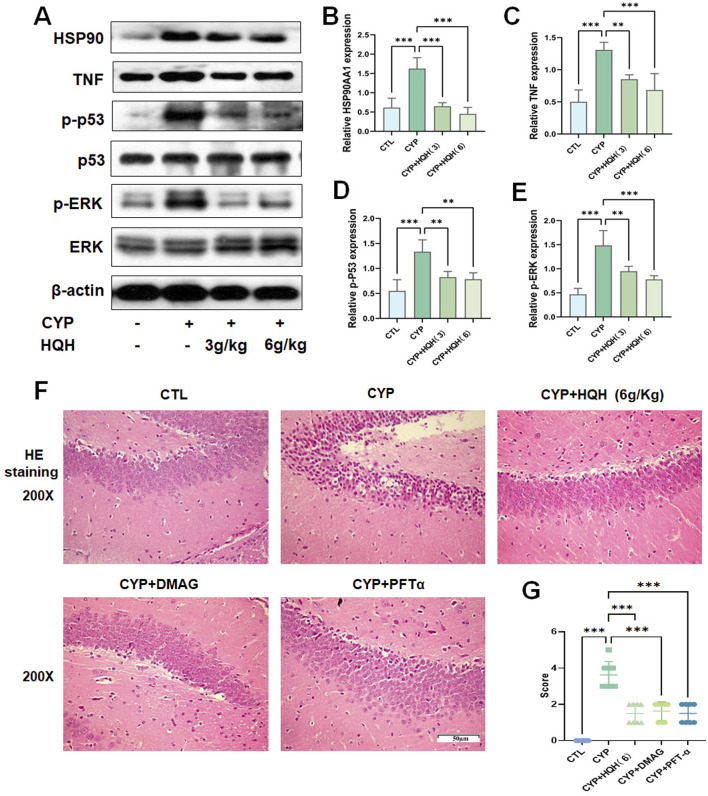
Western blot results showing HQH’s regulatory effects on key protein TNF, ERK, HSP90AA1, p53 in hippocampal tissue **(A–E)**. **(F)** Representative images of hippocampal sections stained with hematoxylin and eosin (H&E) at ×200 magnification. **(G)** Beam Walking Test. **(C–E)** Data are presented as mean ± SD, and statistical significance is indicated by asterisks: ***p < 0.001.

Through molecular docking studies, we identified 4 potential targets related to the neuroprotective effects of HQH on the CYP-treated rat. Western blot analysis was conducted to further verify the changes in 4 proteins in hippocampal tissue). The results showed that compared to the CYP model group, treatment with HQH (6 mg/kg) significantly decreased the protein expression of TNF, HSP90AA1, TP53, MAPK1 (Figures 7A–E). To functionally confirm that HSP90AA1 and TP53 are necessary mediators of HQH protection, rats were co-treated with 17-DMAG (HSP90 inhibitor) or Pifithrin-α (TP53 inhibitor) during CYP exposure, and examined by hippocampal HE staining and beam-walking test. HE staining revealed that 17-DMAG or pifithrin-α treatment significantly attenuated hippocampal neuronal disorganization and nuclear pyknosis, with effects similar to HQH-induced improvement (Figure 7F). Correspondingly, the beam-walking test showed that 17-DMAG or pifithrin-α co-treatment obviously lowered the beam-walking scores compared with the CYP group, reaching levels comparable to the CYP + HQH group (Figure 7G).

## 4 Discussion

Cyclophosphamide-induced neurotoxicity, particularly in the hippocampus, presents a significant challenge in clinical oncology. The present study is the first to comprehensively explore the neuroprotective mechanism of Huaiqihuang (HQH) against CYP-induced hippocampal injury using an integrative approach of network pharmacology, molecular docking, and experimental validation.

We identified 132 overlapping targets between HQH and CYP-induced neurotoxicity. Network pharmacology analysis, GO enrichment, and KEGG pathway enrichment revealed HSP90AA1, TP53, MAPK1, AKT1, RELA, and TNF as top-ranked hub genes. Among these, TNF, HSP90AA1, TP53, and MAPK1, which play crucial roles in cellular responses to stress and injury, showed the strongest binding affinity with CYP in molecular docking simulations, suggesting their central role in CYP-induced neurotoxicity. HSP90, a critical molecular chaperone, maintains cellular homeostasis by assisting in the folding and stabilization of various client proteins (Zang et al., 2025). Its activation in response to CYP-induced neurotoxicity leads to increased production of pro-inflammatory cytokines like TNF, exacerbating neuroinflammation and contributing to neuronal damage. Our findings suggest that HQH exerts its protective effects, in part, by downregulating HSP90, which may disrupt the stability of pro-inflammatory signaling complexes and reduce the activation of MAPK pathways, subsequently decreasing TNF production (Lu et al., 2025; Ahsan Ibrahim et al., 2024; Mengyuan Niu et al., 2022).

MAPK1, a key component of the MAPK cascade, is involved in transducing extracellular signals that regulate inflammatory responses and cell survival (Kundu et al., 2025). Activation of MAPK1 can lead to the phosphorylation and activation of transcription factors that promote inflammation and apoptosis (Liu et al., 2025; Zheng et al., 2025; Cai et al., 2025). Our study indicates that HQH treatment reduces MAPK1 expression, suggesting that it may counteract CYP-induced inflammation and apoptosis by inhibiting MAPK signaling.

TP53, known as the “guardian of the genome,” is a central tumor suppressor that regulates cellular responses to DNA damage, including apoptosis, cell cycle arrest, and DNA repair (Holoubek et al., 2025; Lin et al., 2025; Luo et al., 2025). In the context of CYP-induced neurotoxicity, TP53 activation can promote neuronal apoptosis, contributing to hippocampal damage. The significant decrease in TP53 expression observed with HQH treatment implies that HQH may protect neurons by inhibiting TP53-mediated apoptotic pathways, thus preserving neuronal viability.

The interactions between HSP90, TP53, MAPK1, and TNF are part of a complex network that is central to the cellular response to oxidative stress, a hallmark of CYP-induced neurotoxicity. Oxidative stress can activate MAPK and NF-κB pathways, leading to the upregulation of TNF and other inflammatory cytokines (Zhang et al., 2025; Da et al., 2025; He et al., 2025). By targeting HSP90, HQH may modulate the redox balance maintained by these pathways, thereby reducing oxidative stress and its downstream effects on inflammation and apoptosis.

Our findings indicate that HQH exerts potent neuroprotective effects by mitigating oxidative stress, inhibiting apoptosis, and modulating inflammatory signaling. Specifically, CYP administration disrupted redox homeostasis, activating MAPK and NF-κB signaling cascades, leading to neuronal apoptosis and neuroinflammation. HQH intervention effectively reversed these alterations, consistent with previous reports of its antioxidant and anti-inflammatory properties.

Overall, HQH targets multiple pathological processes, underscoring its potential as a multitarget neuroprotective agent. By regulating key proteins like HSP90AA1, HQH modulates both oxidative stress and inflammatory pathways, providing a mechanistic basis for its therapeutic application in the prevention and treatment of chemotherapy-induced neurotoxicity.

## 5 Conclusion

This study provides compelling evidence that Huaiqihuang (HQH) offers significant neuroprotection against cyclophosphamide (CYP)-induced hippocampal neurotoxicity. By integrating network pharmacology, molecular docking, and experimental validation, we identified key targets and pathways involved in oxidative stress, apoptosis, and neuroinflammation that mediate HQH’s protective effects. Specifically, HQH modulates the NF-κB and MAPK signaling pathways, demonstrating its potential as a multitarget therapeutic. These findings lay a solid scientific foundation for the clinical application of HQH as a complementary strategy to prevent or mitigate chemotherapy-induced neurotoxicity and improve patient outcomes.

## Data Availability

The raw data supporting the conclusions of this article will be made available by the authors, without undue reservation.
